# Brace *versus* cast following surgical treatment of distal radial fracture: a prospective randomised study comparing quality of recovery

**DOI:** 10.12688/f1000research.52046.2

**Published:** 2022-01-28

**Authors:** Irén Sellbrant, Johanna Blomstrand, Jon Karlsson, Bengt Nellgård, Jan Jakobsson

**Affiliations:** 1 Department of Anaesthesiology and Intensive Care, Institute of Clinical Science, Sahlgrenska Academy, University of Gothenburg, Sahlgrenska University Hospital, Gothenburg, 431 30, Sweden; 2 Department of Orthopaedics, Institute of Clinical Science, Sahlgrenska Academy, University of Gothenburg, Sahlgrenska University Hospital, Gothenburg, 431 30, Sweden; 3 Department of Anaesthesia & Intensive Care, Institute of Clinical Science, Karolinska Institute, Danderyd University Hospital, Stockholm, 182 88, Sweden

**Keywords:** brace, cast, distal radial fracture, immobilisation after surgery, postoperative oxycodone consumption, quality of recovery, QoR-15, removable splint.

## Abstract

**Background**: Immobilisation following surgical treatment of distal radial fractures (DRF) is traditionally performed with a dorsal cast splint. There is an interest in changing the rigid cast to a removable brace. This can reduce the risk for cast-corrections, complications and improve recovery of function.

The aim of the study was to compare quality of recovery (QoR) between brace and traditional cast for immobilisation during the first postoperative week.

**Methods: **60 patients with American Society of Anesthesiologists (ASA) physical status 1–3, scheduled for surgical treatment of DRF under a supraclavicular block (SCB) in a day-surgery setting were randomised into two groups of immobilisation post-surgery; brace (n=30) *versus* traditional cast (n=30). Study objectives were: differences in self-assessed QoR using the QoR-15 questionnaire, postoperative oral oxycodone consumption, perioperative time events and unplanned healthcare contacts one week postoperatively.

**Results**: 54 patients, 46 females/eight males were included in the analysis; 27 with brace and 27 with traditional cast. QoR-15 median scores improved significantly from baseline/preoperative to day 7 (brace p=0.001, cast p=0.001) with no differences between the two groups. The only difference found was that patients in the brace group had significantly worse pain score 24-hours post-surgery (p=0.022). No significant differences were seen in total median oxycodone consumption the first three postoperative days. No differences were found in perioperative events or unplanned healthcare contacts.

**Conclusions**: Brace appears to be a feasible option to traditional cast for immobilisation following surgical treatment of DRF. The early QoR was similar in both groups apart from more pain in the brace group the first 24 postoperative hours.

## Introduction

Distal radial fracture (DRF) is one of the most common fractures. ^1^ Fracture reduction and cast treatment is the common practice; however, if good fracture positioning cannot be achieved, open surgical correction and fixation is the most preferred option. Surgical treatment of DRF is commonly performed under either regional anaesthesia (RA) like a supraclavicular block (SCB) or general anaesthesia (GA). The currently preferred surgical treatment is volar-plate fixation followed by external cast application. ^2^ The procedure is usually associated with moderate postoperative pain, although occasionally severe. Poor postoperative pain control may interfere with rehabilitation, delay recovery and adversely affect outcomes. Achieving early pain control may help to improve patient satisfaction and functional outcomes. The cast is usually changed to a removable and adjustable brace after two weeks. Rehabilitation support from an occupational therapist is valuable in order to achieve the best final functional outcome. The use of a brace for stabilisation following DRF has now gained increased interest, and studies have shown high patient acceptance. ^3- 5^ There is, however, sparse information on the effect of the use of brace following surgical treatment of DRF in adult patients. ^6, 7^

Self-assessed quality of recovery (QoR) questionnaires evaluating the postoperative course in a multi-dimensional perspective have gained increasing interest. To capture the QoR from the patients’ perspective, a variety of QoR instruments have been developed. ^8- 14^ One of these rating scales, the 40-item QoR-40, has been most extensively validated and demonstrates excellent psychometric properties. It has been translated and validated in different languages. Quality of recovery-15 (QoR-15) was developed to assess the recovery in a more simplified and user-friendly manner without reducing the quality of the instrument. It is a unidimensional measurement of QoR assessing five domains: pain, physical well-being, physical independence, psychological support and emotional state. ^15^ Assessing perioperative interventions by means of QoR could provide a broader evaluation than merely recording the pain and analgesia consumption. Chazapis *et al* showed that QoR-15 is an acceptable and feasible outcome measure for day-surgery patients. ^16^ Lyckner *et al* translated and culturally adapted the QoR-15 into Swedish and scores demonstrated acceptable validity, reliability and responsiveness; it can be performed in 3 minutes. ^17^

The aim of the present study was to assess QoR and opioid consumption, following surgical treatment of distal radial fractures (DRF) comparing postoperative immobilisation with brace *versus* traditional cast, during the first postoperative week.

We hypothesised that a brace, applied directly after surgery, will give the same or better QoR as a traditional cast during the first postoperative week after surgical treatment of DRF in day surgery.

## Methods

This randomised study is a part of a randomised clinical trial approved by the Gothenburg Ethical Committee (May 31 2018; registration no 214-18). It was also registered in the Sahlgrenska University Hospital GDPR (General Data Protection Regulation) database on August 28, 2018. The study was conducted in accordance with the tenets of the 1964 Declaration of Helsinki. It was retrospectively registered in clinicaltrials.gov (NCT03749174) on November 21, 2018 with explicit information about start of patient inclusion: September 3, 2018. All patients aged between 18-78 years, with American Society of Anesthesiologists (ASA) physical status 1–3 and scheduled for day surgery of a DRF between September 3, 2018, and June15, 2020, at the Department of Anaesthesia and Intensive Care, Sahlgrenska University Hospital/Mölndal Hospital, Gothenburg, Sweden, were assessed for eligibility. Written informed consent with permission to publish was obtained from all patients before enrollment.

Opioid-naïve patients were included; when having a closed DRF assessed on radiographs and classified as AO 23 A-C1 (Orthopaedic Trauma Association), ≤17 days from trauma and scheduled for operative fixation with a locked volar-plate. Finally, maximum length-of-surgery had to be <90 min and all surgeons used tourniquet. Exclusion criteria were: multifractures, inflammatory diseases, dementia, severe psychiatric disorder or other cognitive dysfunction, ongoing drug and alcohol abuse, known local anaesthetic allergy, pregnancy and finally, no fluency of the Swedish language.

Fracture classification was performed by an experienced orthopaedic surgeon.

In total, 142 patients were informed about the study. 22 patients declined to participate, leaving 120 patients to be included following written informed consent. The effect of anaesthetic technique was assessed in 90 patients and is presented separately elsewhere. The patients in this part of the study were randomised to different anaesthetic techniques, SCB with mepivacaine, SCB with ropivacaine or general anaesthesia. Rebound pain, difference in pain (NRS) at rest at 24-hours and further during the first three days after surgery between the short acting block (mepivacaine) and long acting block (ropivacaine), with general anesthesia being control group was studied. 60 patients with the same anaesthetic technique (SCB with mepivacaine) from this study were randomised by the investigator, using sequentially numbered opaque envelopes, to one of two groups of immobilisation methods post-surgery: 1) traditional dorsal cast (n = 30); and 2) removable brace (n = 30), prefabricated and stabled with volar and dorsal steel rails (Wrist lacer, Camp Scandinavia AB).

All patients followed the dedicated day-care bundle for enhanced recovery and safe discharge on the day of surgery. Perioperative care was optimised for providing rapid and effective recovery.

The QoR was measured with the QoR-15 score, (see the extended data). ^17^ The questionnaire uses an 11-point numerical rating scale (for positive items, 0 = “none of the time” to 10 = “all of the time”; for negative items the scoring is reversed; maximum score 150). The 11-point numerical rating scale leads to a minimum score of 0 (very poor recovery) and a maximum score of 150 (excellent recovery). Question 7: “Getting support from hospital” was not useful in this study since all patients were in a day-surgery setting and followed-up with telephone calls. That reduced the maximum total score to 140 points instead of 150. The five dimensions of health were incorporated in the 14 questions (questions 1–6 and 8–15): physical comfort (questions 1–4 and 13), physical independence (questions 5 and 8), psychological support (question 6), pain (questions 11 and 12) and emotions (questions 9, 10, 14, and 15).

All patients received oral premedication, acetaminophen (1000 mg), oxycodone (5 or 10 mg; 5 mg to >70 years and/or <60 kg), etoricoxib (90 mg; if no contraindication) and meclizine (25 mg). All patients were given 8 mg betamethasone *intravenous* perioperatively.

All patients underwent surgical treatment with a volar-plate fixation by a senior orthopaedic surgeon and were then immobilised for two weeks with one of the two randomised immobilisation methods applied directly post-surgery. After two weeks, the patients allocated to the traditional cast had this routinely replaced with a brace. All patients obtained a SCB with a short-acting local anaesthetic agent (mepivacaine 1%, 25-30 mL) and were offered a mild sedation with propofol perioperatively. SCB was performed by an experienced anaesthesiologist with ultrasound guidance.

Patients being considered stable and adequate pain-relieved bypassed the Post-Anaesthesia Care Unit (PACU) and were transferred directly to the post-surgery ward. Patients needing monitoring stayed in the PACU until considered stable. All patients were planned to be discharged home from the step-down ward.

Patients obtained a protocol to note the type, dose and frequency of analgesic consumption at home and they all received the same postoperative pain management at discharge: oxycodone 5–10 mg and acetaminophen 1000 mg for pain control, respectively. They received a prescription of these medications to be taken *ad libitum* within a daily maximum dose of 30–40 mg oxycodone and 4000 mg of acetaminophen.

At day 2–4 postoperatively, the patients had their first appointment to the occupational therapist with following appointments scheduled at 2, 6, 12 weeks and 1 year postoperatively.

## Data collection

All data were collected by the same two investigators, while patients were still in hospital and then by four follow-up telephone calls at 24, 48, 72 hours and 7 days after discharge. Patient characteristics were collected prior to start of surgery and anaesthesia. QoR-15 was performed four times (baseline/preoperatively, 24, 72 hours and 7 days post-surgery). The pain domain was used for the assessment of pain. Postoperatively, we collected oral oxycodone consumption, administered at the hospital and after discharge; the first 3 postoperative days and 7 days after surgery. We also registered perioperative time events; surgery time (including fixation with cast or brace), time the anaesthetic nurse was occupied with the patient, theatre time, unplanned admission, number of patients needing PACU stay, total time in hospital, SCB total duration time and duration time after surgery.

The primary outcome was difference in the sum median (interquartile range) and its five domains of QoR-15 score at baseline, 24 hours, 72 hours and 7 days after surgery between the two groups.

The secondary outcome was the total median (interquartile range) amount of postoperative oral oxycodone consumption administered at the hospital and after discharge, the first 3 postoperative days and 7 days after surgery. Finally, perioperative time events were as described above.

### Statistical analyses

Numerical data are presented as mean and standard deviation (SD) and median and quartiles for non-normally distributed data. Categorical data is presented as numbers and percent. Differences between the study groups, brace and cast, were studied with independent sample t-test for normally distributed data and Mann–Whitney U-test for skewed data. Differences in proportion were studied with a Chi-squared test. The QoR data are presented as median and interquartile range (IQR). Differences between median QoR-15 and changes in QoR-15 score between time points were analysed with non-parametric tests, Mann–Whitney U-test and Kruskal–Wallis test as applicable. A p < 0.05 was considered statistically significant.

## Results

**Figure 1.**
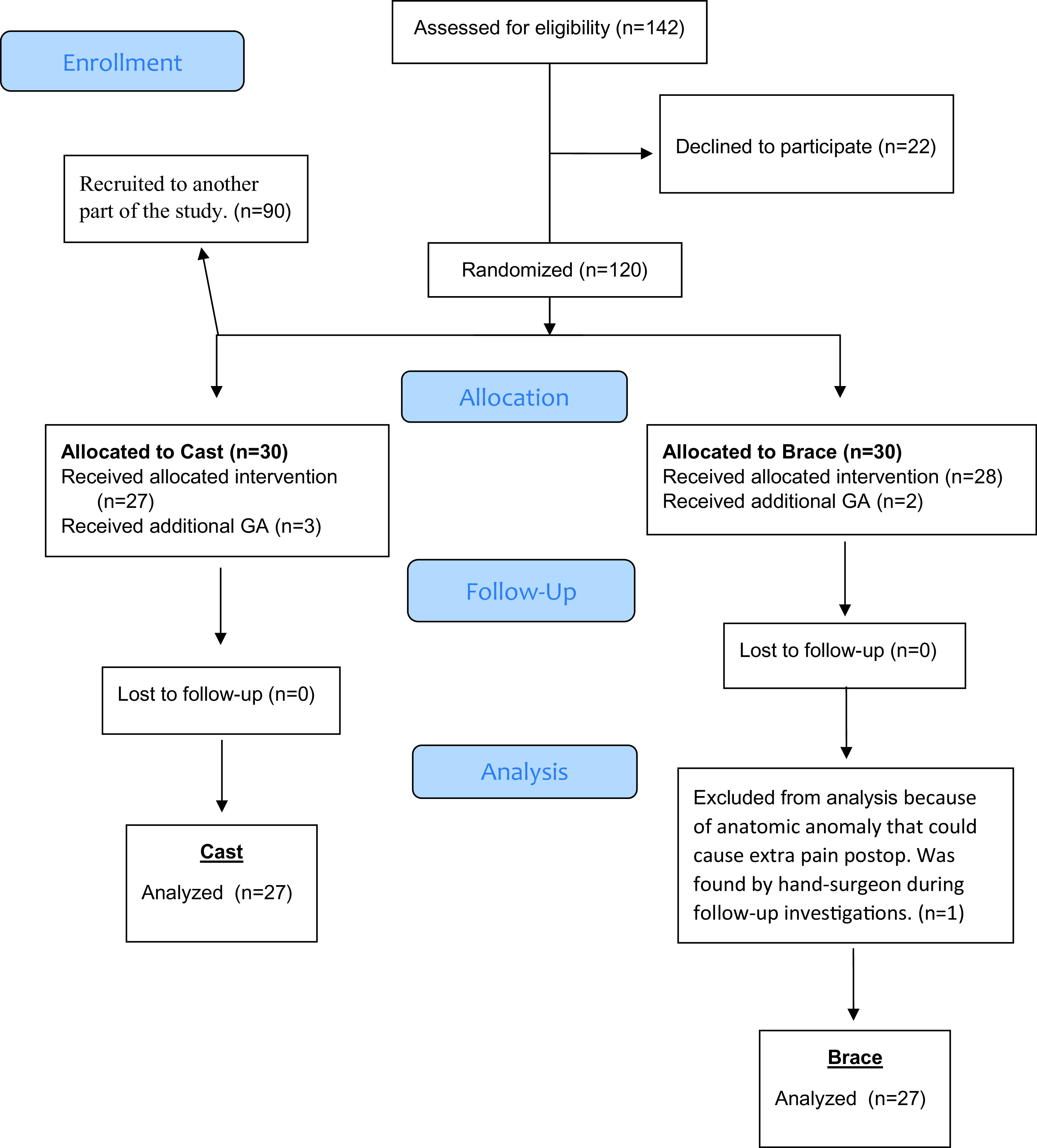
CONSORT Flowchart. Flow of patients through the trial. General anesthesia *(GA)*.

60 patients scheduled for surgical treatment of DRF were included in the study following informed consent. Six patients were not included in the analysis; five due to failed supraclavicular block and one because of anatomic anomaly ( Figure 1).

54 patients, 46 females and eight males with a mean age of 56 (SD ± 15) years, ASA 1–3 patients, were included in the analysis, 27 patients in each group; brace *versus* cast for immobilisation post-surgery. The mean age was lower (p = 0.02) and the mean BMI was higher (p = 0.02) in the brace group. No further differences were found in patients’ baseline characteristics ( Table l).

**Table 1.** Patient characteristics and clinical data presented as mean (±2 SD) or absolute number as appropriate. Classification of patients´ health and comorbidity level by the American Society of Anesthesiologists *(ASA)* system, Body mass index *(BMI)*, Apfel score; riskfactors (1-4) for PostOperative Nausea and Vomiting *(PONV).*

*Characteristics*	Cast (n = 27)	Brace (n = 27)	p-value
***Age*** ***(years)***	60 (± 10)	51 (± 17)	p = 0.02
***Gender; female/male*** ***(no of patients)***	24/3	22/5	0.44
***BMI*** ***(kg m*** ^***−2***^ ***)***	23 (± 2.3)	25 (± 3.5)	p = 0.02
***Smoking; yes/no*** ***(number of patients)***	3/24	4/23	0.69
***Snuffing; yes/no*** ***(number of patients)***	2/25	1/26	0.55
***Apfel score; 1/2/3/4*** ***(number of patients)***	0/4/17/6	0/7/14/6	0.58
***ASA; 1/2/3*** ***(number of patients)***	13/13/1	14/13/0	0.60
***Day from injury*** ***Number of days)***	8.4 (± 3.1)	9.7 (± 3.6)	0.15

Time from injury and perioperative time events did not differ between the groups. The majority of patients could bypass the PACU and were transferred directly to the step-down ward. The SCB resolution-time was a mean of 2.6 hours after surgery in both groups. No further differences were seen between the groups in any perioperative time events ( Table 2).

**Table 2.** Perioperative time observations. Data are presented as mean (2 ±SD) or for categorical data (no; %). Post Anaesthesia Care Unit ( *PACU*), Day Surgery *(DS)*, Supraclavicular block *(SCB*) *.*

*Perioperative time events*	Cast (n = 27)	Brace (n = 27)	p-value
***Anaesthesia nurse time (minutes)***	152 (±46)	155 (±47)	0.97
***Theater time (minutes)***	192 (±48)	172 (±43)	0.11
***Surgery time (including plaster/orthosis) (minutes)***	71 (±21)	69 (±20)	0.48
***PACU admitted patients (number of patients, %)***	4 (15%)	1 (4%)	
***Unplanned Admission (number of patients, %)***	2 (7.4%)	0	
***Hospital time, DS patients (minutes)***	501 (±100)	503 (±79)	0.99
***SCB total duration time (hours)***	4.6 (±1.1)	4.9 (±1.5)	0.78
***SCB duration time after surgery (hours)***	2.7 (±1.0)	2.6 (±1.5)	0.55

### Unplanned admission and unplanned healthcare contacts after discharge

Two patients (7.4%) from the cast group were admitted overnight post-surgery, both due to social reasons. None of the patients in the brace group needed an unplanned overnight admission and neither did any patient need unplanned healthcare contacts after hospital discharge during the first postoperative week.

### Quality of recovery

**Figure 2.**
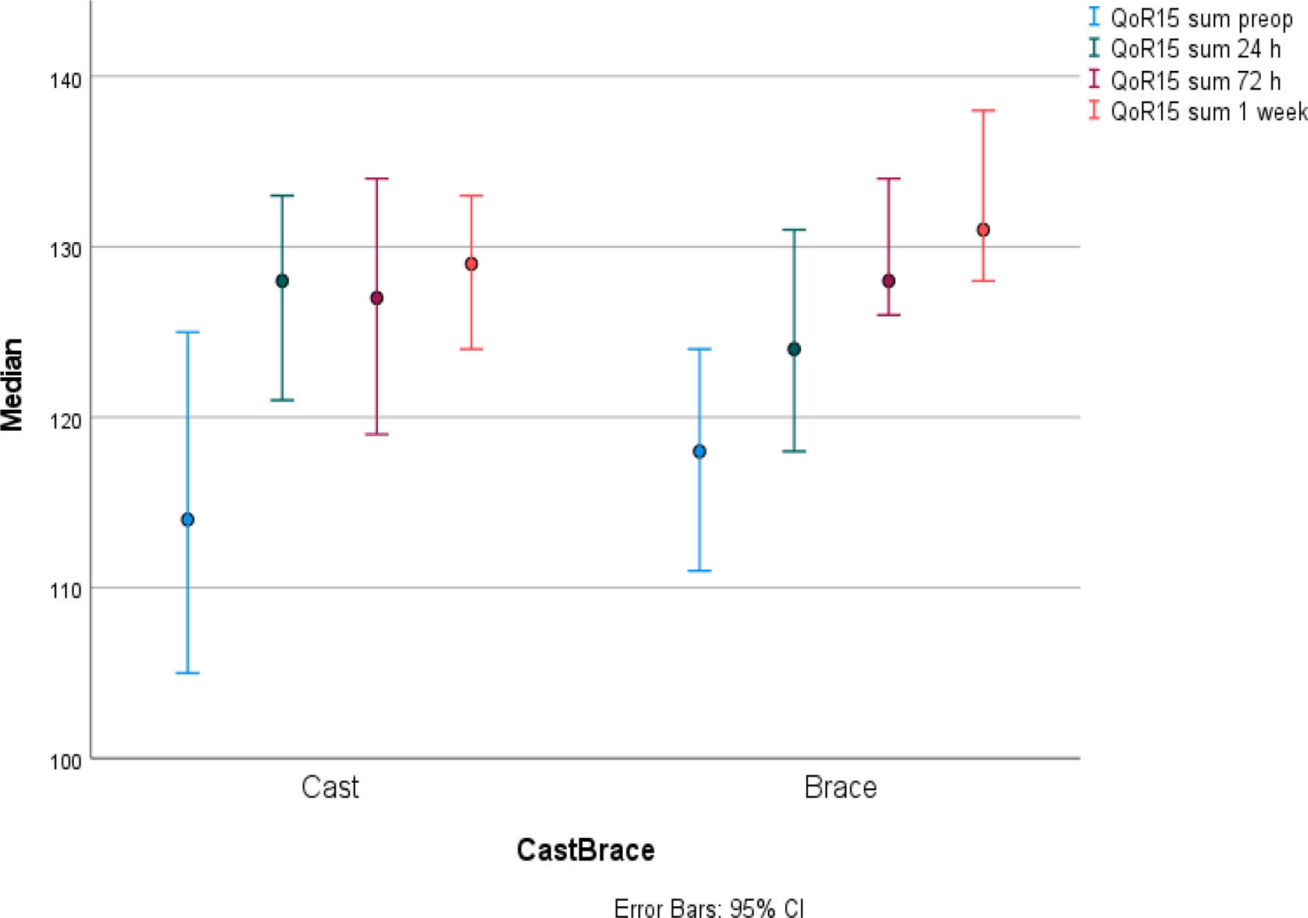
Quality of Recovery -15 ( *QoR-15)* presented in median *(IQR)* for cast and brace groups at each time point. Maximum score possible was 140 points. Both groups showed an increase in median QoR-15 score from baseline and up to 1-week post-surgery (cast p = 0.001, brace p = 0.001).

The median QoR-15 score was equal in the two groups at preoperative baseline assessment. Then, there were an increase in median QoR-15 score in both study groups from baseline and up to 1 week after surgery (cast p = 0.001, brace p = 0.001). No postoperative reduction in median QoR-15 score was seen. There was a slightly different pattern between the two groups and thus, the cast group had the largest increase from baseline already after 24 hours while the brace group increased slower and reached the largest increase from baseline first at 72 hours post-surgery. The median QoR-15 score did not differ between the groups one week postoperatively ( Figure 2, Table 3).

**Table 3.** Quality of Recovery -15 ( *QoR-15)* and the sum for the five different domains (with maximum score possible in parentheses) are presented in median *(IQR)* at the different time-points.

QoR-15 score, *(max points)*	Cast (n = 27)	Brace (n = 27)	p-value
***Preoperative, baseline (140p)*** *Summary score*	114 (104-125)	118 (109-125)	0.34
*Pain (20p)*	15 (10-19)	17 (15-17)	0.19
*Physical comfort (50p)*	44 (39-48)	46 (44-50)	0.048
*Physical Independency (20p)*	15 (12-16)	16 (14-16)	0.97
*Psychological support (10p)*	10 (10-10)	10 (10-10)	0.53
*Emotional state (40p)*	33 (20-38)	32 (27-36)	0.92
***Postoperative, 24 hours (140p)*** *Summary score*	128 (118-133)	124 (118-132)	0.31
*Pain (20p)*	18 (14-20)	15 (10-18)	0.022
*Physical comfort (50p)*	49 (46-50)	48 (45-50)	0.60
*Physical Independency (20p)*	16 (12-18)	16 (12-18)	0.99
*Psychological support (10p)*	10 (10-10)	10 (10-10)	0.15
*Emotional state (40p)*	38 (35-40)	38 (36-40)	0.79
***Postoperative, 72 hours (140p)*** *Summary score*	127 (118-135)	128 (125-134)	0.72
*Pain (20p)*	18 (15-20)	18 (16-19)	0.97
*Physical comfort (50p)*	47 (44-50)	49 (46-50)	0.28
*Physical Independency (20p)*	15 (15-18)	17 (14-18)	0.77
*Psychological support (10p)*	10 (10-10)	10 (10-10)	1.0
*Emotional state (40p)*	38 (35-40)	38 (35-40)	0.90
***Postoperative, 1 week (140p)*** *Summary score*	129 (123-134)	131 (128-138)	0.20
*Pain (20p)*	19 (17-20)	20 (17-20)	0.38
*Physical comfort (50p)*	49 (44-50)	49 (47-50)	0.52
*Physical Independency (20p)*	16 (14-18)	18 (15-18)	0.40
*Psychological support (10p)*	10 (10-10)	10 (10-10)	0.32
*Emotional state (40p)*	37 (32-40)	38 (36-40)	0.70

**Figure 3.**
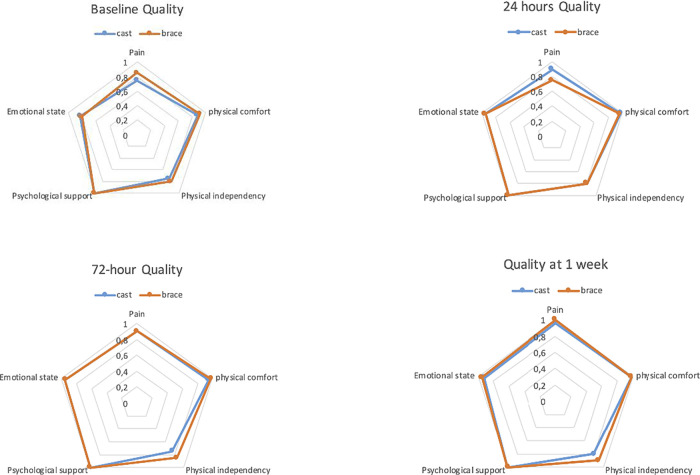
The Spider-chart shows the five domains median scores put in perspective of each domains max-score between groups over time.

The preoperative/baseline QoR-15 score showed no difference between the groups. However, the domain physical comfort was significantly lower (p = 0.048) in the cast group. The different domains in the QoR-15 questionnaire showed only minor differences between the two groups at the four time-points studied. There was a significant difference in pain-score at 24 hour-assessment when brace scored worse than cast (p = 0.022) ( Figure 3, Table 3).

### Postoperative oral oxycodone consumption

Median postoperative oral oxycodone consumption was low overall. Median consumption for both groups together was 27.7 (IQR 9-46) mg the first three postoperative days. No differences in oxycodone consumption were seen ( Table 4).

**Table 4.** Postoperative oral oxycodone consumption in mg presented in median *(IQR)* at different time-points.

*Postoperative oral Oxycodone consumption in mg*	Cast (n = 27)	Brace (n = 27)	p-value
***Step-down ward***	5 (0-5)	5 (5-5)	0.66
***Discharge - 24 h***	5 (0-10)	10 (10-20)	0.08
***24 – 48 h***	5 (0-10)	5 (0-20)	1.0
***48 – 72 h***	0 (0-10)	0 (0-10)	1.0
***Sum oxycodone day 0-3***	15 (5-40)	35 (15-50)	0.27
***Day 7***	0 (0-0)	0 (0-0)	0.47
***Sum oxycodone day 0-3+7***	20 (5-40)	35 (15-50)	0.27

## Discussion

This study was initiated to assess the feasibility to use a removable brace compared with a traditional cast following surgical treatment of distal radial fracture (DRF). There are two main findings in the present study:

Firstly, supported our hypothesis, we found no significant difference in the QoR-15 score, a difference was seen in subdomain pain, noted at 24 hours, between the brace and cast groups during the first postoperative week. The numerical difference is lower than what has been shown of clinical relevance. ^18^ The multifactorial reasons of the experienced pain have not been furthermore investigated in this study.

Secondly, we found no deterioration in the median QoR-15 score post-surgery in neither of the two groups. The postoperative median QoR-15 score increased over time from baseline until one-week post-surgery in both study groups, with no differences at any time-point assessed. This is a scarce finding since most patients usually do show impairment at 24 hours post-surgery. ^16^ This could indicate that the preoperative QoR-15 score may not be a true baseline score. Focusing on individual items of the scores, the results indicated that patients were tired, anxious and in pain 24 hours prior to surgery. Further, all the patients in the present study had their trauma in average 9 days prior to surgery and some of them had been treated conservatively without success. These circumstances may not provide an ideal baseline for assessing recovery following surgery and could explain the impaired QoR-15 baseline scores; however, it may still constitute a baseline for comparison. This can thus explain the result that we found no postoperative deterioration in the median QoR-15 score at 24 hours post-surgery, merely a continuous improvement. Chazapis *et al.* had similar result in their study assessing orthopaedic patients scheduled for day surgery (DS). ^16^

Both study groups had a SCB with a short-acting local anaesthetic agent (mepivacaine) as part of a multi-modal analgesia concept, reducing the risk for rebound pain after discharge. ^19^ Oral pain therapy was started before surgery, where all patients had acetaminophen, etoricoxib, oxycodone given preoperatively and 8 mg betamethasone given *intravenously* perioperatively as a part of the multi-modal analgesic treatment. Thus, the difference in early pain is possibly related to the brace. The numerically higher oxycodone consumption, though not statistically significant, may at least partly be associated to oxycodone dosage, adjusted to age and BMI, since patients with brace were both significantly younger and had higher BMI. No further differences were seen in the patients’ self-assessed QoR or oxycodone consumption.

Day surgery (DS) continues to grow as a field of perioperative care and now also subacute surgeries, like fractures, are performed as day surgery. None of the patients in the present study required hospital admission. Two patients were however admitted of social reasons, not related to recovery. There were no unplanned healthcare contacts during the first postoperative week and therefore, this study confirms the feasibility of scheduling surgical treatment of DRF as a day surgery procedure. The immobilisation techniques hardly impact day surgery planning, nor may it affect theatre times. Although we found no time gain in the brace-group, we speculate that using a brace should reduce time in theatre as we avoid the time for cast construction. Every intervention that could facilitate a rapid, safe and effective patient turnover is of importance.

The ability to resume normal activities of daily living after surgery and anaesthesia is an important indicator of a high quality of perioperative care. Most patients’ QoR-15 scores had returned to their preoperative values and exceeded them after 24 hours. This randomised study suggests that measurement of QoR-15 before surgery, (but not on the day of surgery), and 48 h postoperatively could provide a useful and feasible assessment of patient-reported quality of recovery after day case orthopaedic surgery. ^16^

### Strengths and limitations

This study had a prospective randomised clinical design that minimised the risk of confounding factors. It was a single-centre design without any loss to follow-up reducing the risk of selection and information bias and also warrants generalisability of this study. Only two investigators collected the data ensuring consistency and high standard of data collection.

However, there are some limitations to this study. All results must be assessed remembering the fact that no sample size calculation was made for this particular study. The study is a part of a larger study assessing anaesthetic techniques and the sample size calculation was made for that study.

The aim of QoR-15 was to assess QoR following anaesthesia and surgery and not to assess QoR following different immobilisation techniques. The sensitivity of the QoR-15 instrument may have been too low for this intervention and a potential ceiling effect must be acknowledged. Moreover, the trial was not blinded to any of the anaesthesia/surgery staff, nor to the study nurse and nor to the patient. We excluded patients with poor Swedish comprehension and severe pre-existing medical conditions. We cannot disregard the fact, that a nurse repeatedly calling the patients to ask for their health status during the first postoperative week could contribute to a therapeutic effect.

## Conclusion

We found brace, applied directly after surgery, to be a feasible option to traditional cast for immobilisation following surgical treatment of DRF. There was no difference in quality of recovery, assessed by QoR-15, during the first postoperative week, between brace and cast. The effects of brace immobilisation on more protracted recovery and more complex fractures needs further studies.

## Data Availability

OSF: Underlying data for ‘Brace *versus* cast following surgical treatment of distal radial fracture: a prospective randomised study comparing quality of recovery’, https://doi.org/10.17605/OSF.IO/KJC2N. ^20^ This project contains the following underlying data: pain scores, oxycodone consumption and QoR15 scores Data are available under the terms of the Creative Commons Attribution 4.0 International license (CC BY 4.0). OSF: CONSORT checklist for ‘Brace *versus* cast following surgical treatment of distal radial fracture: a prospective randomised study comparing quality of recovery’, https://doi.org/10.17605/OSF.IO/KJC2N. ^20^ Data are available under the terms of the Creative Commons Attribution 4.0 International license (CC BY 4.0).

## References

[1] Jerrhag D Englund M Karlsson MK : Epidemiology and time trends of distal forearm fractures in adults - a study of 11.2 million person-years in Sweden. *BMC Musculoskelet Disord.* 2017;18(1):240. 10.1186/s12891-017-1596-z 28576135 PMC5457562

[2] Mellstrand-Navarro C Pettersson HJ Tornqvist H : The operative treatment of fractures of the distal radius is increasing: results from a nationwide Swedish study. *Bone Joint J.* 2014;96-b(7):963–9. 10.1302/0301-620X.96B7.33149 24986952

[3] McVeigh KH Berger TG Cudahy R : An Evidence-Based Approach to Casting and Orthosis Management of the Pediatric, Adolescent, and Young Adult Population for Injuries of the Upper Extremity: A Review Article. *Clin J Sport Med.* 2019. 10.1097/JSM.0000000000000718 30730385

[4] Cui Z Yu B Hu Y : Dynamic versus static external fixation for unstable distal radius fractures: an up-to-date meta-analysis. *Injury.* 2012;43(7):1006–13. 10.1016/j.injury.2011.11.018 22178307

[5] Foster BD Sivasundaram L Heckmann N : Distal Radius Fractures Do Not Displace following Splint or Cast Removal in the Acute, Postreduction Period: A Prospective. *Observational Study. J Wrist Surg.* 2017;6(1):54–9. 10.1055/s-0036-1588006 28119796 PMC5258127

[6] Stuby FM Döbele S Schäffer SD : Early functional postoperative therapy of distal radius fracture with a dynamic orthosis: results of a prospective randomized cross-over comparative study. *PLoS One.* 2015;10(3):e0117720. 10.1371/journal.pone.0117720 25822197 PMC4378993

[7] Handoll HH Huntley JS Madhok R : Different methods of external fixation for treating distal radial fractures in adults. *Cochrane Database Syst Rev.* 2008(1):Cd006522. 10.1002/14651858.CD006522.pub2 18254105 PMC8925648

[8] Gornall BF Myles PS Smith CL : Measurement of quality of recovery using the QoR-40: a quantitative systematic review. *Br J Anaesth.* 2013;111(2):161–9. 10.1093/bja/aet014 23471753

[9] Stark PA Myles PS Burke JA : Development and psychometric evaluation of a postoperative quality of recovery score: the QoR-15. *Anesthesiology.* 2013;118(6):1332–40. 10.1097/ALN.0b013e318289b84b 23411725

[10] Myles PS Hunt JO Nightingale CE : Development and psychometric testing of a quality of recovery score after general anesthesia and surgery in adults. *Anesth Analg.* 1999;88(1):83–90. 10.1097/00000539-199901000-00016 9895071

[11] Royse CF Newman S Chung F : Development and feasibility of a scale to assess postoperative recovery: the post-operative quality recovery scale. *Anesthesiology.* 2010;113(4):892–905. 10.1097/ALN.0b013e3181d960a9 20601860

[12] Royse CF Williams Z Purser S : Recovery after nasal surgery vs. tonsillectomy: discriminant validation of the Postoperative Quality of Recovery Scale. *Acta Anaesthesiol Scand.* 2014;58(3):345–51. 10.1111/aas.12264 24417321

[13] Royse CF Williams Z Ye G : Knee surgery recovery: Post-operative Quality of Recovery Scale comparison of age and complexity of surgery. *Acta Anaesthesiol Scand.* 2014;58(6):660–7. 10.1111/aas.12273 24571268

[14] Lindqvist M Granstrom A Schening A : Cognitive testing with the Post-Operative Quality of Recovery Scale in pre-surgery cancer patients--a controlled study. *Acta Anaesthesiol Scand.* 2015;59(6):763–72. 10.1111/aas.12473 25969870

[15] Kleif J Waage J Christensen KB : Systematic review of the QoR-15 score, a patient- reported outcome measure measuring quality of recovery after surgery and anaesthesia. *Br J Anaesth.* 2018;120(1):28–36. 10.1016/j.bja.2017.11.013 29397134

[16] Chazapis M Walker EM Rooms MA : Measuring quality of recovery-15 after day case surgery. *Br J Anaesth.* 2016;116(2):241–8. 10.1093/bja/aev413 26787793

[17] Lyckner S Boregard IL Zetterlund EL : Validation of the Swedish version of Quality of Recovery score-15: a multicentre, cohort study. *Acta Anaesthesiol Scand.* 2018;62(7):893–902. 10.1111/aas.13086 29417552

[18] Myles PS , Myles DB. An Updated Minimal Clinically Important Difference for the QoR‐15 Scale. *Anesthesiology.* 2021;135(5):934–5. 34543410 10.1097/ALN.0000000000003977

[19] Lavand'homme P : Rebound pain after regional anesthesia in the ambulatory patient. *Curr Opin Anaesthesiol.* 2018. 10.1097/ACO.0000000000000651 30124544

[20] Jakobsson J : Brace vs cast. 2021, April 14. 10.17605/OSF.IO/KJC2N

