# Supplementary material for: Brace versus cast following surgical treatment of distal radial fracture: a prospective randomised study comparing quality of recovery
Source: F1000Res. 2022 Jan 28;10:336. Originally published 2021 May 4. [Version 2] doi: 10.12688/f1000research.52046.2 (PMC8837810; doi:10.12688/f1000research.52046.2)
Supplement: Peer review history

# Peer review: Reviewer response for version 2

**DOI:** 10.5256/f1000research.120605.r121450

**Published:** 2022-02-11

**Authors:** Jakob Walldén

**Affiliations:** 1 Department of Surgical and Perioperative Sciences, Anaesthesiology and Intensive Care Medicine (Sundsvall), Umeå University, Sundsvall, Sweden

The authors have addressed the issues raised in the peer review report and have made changes and clarifications in the manuscript. I have no further questions to this well-written manuscript.

Is the work clearly and accurately presented and does it cite the current literature?

Yes

If applicable, is the statistical analysis and its interpretation appropriate?

Yes

Are all the source data underlying the results available to ensure full reproducibility?

Yes

Is the study design appropriate and is the work technically sound?

Yes

Are the conclusions drawn adequately supported by the results?

Yes

Are sufficient details of methods and analysis provided to allow replication by others?

Yes

Reviewer Expertise:

Anesthesiology, postop-recovery

I confirm that I have read this submission and believe that I have an appropriate level of expertise to confirm that it is of an acceptable scientific standard.

# Peer review: Reviewer response for version 2

**DOI:** 10.5256/f1000research.120605.r121451

**Published:** 2022-01-31

**Authors:** Robert Gvozdenovic

**Affiliations:** 1 Department of Orthopedic Surgery, Hand Surgery Unit, Copenhagen University Hospital Gentofte, Hellerup, Denmark

I am happy with the answers and comments provided by the corresponding author of this paper. All my questions and suggestions have been profoundly answered, and accordingly, some changes has been added in the manuscript. I have no further comments on this work.

Is the work clearly and accurately presented and does it cite the current literature?

Yes

If applicable, is the statistical analysis and its interpretation appropriate?

Yes

Are all the source data underlying the results available to ensure full reproducibility?

Yes

Is the study design appropriate and is the work technically sound?

Yes

Are the conclusions drawn adequately supported by the results?

Yes

Are sufficient details of methods and analysis provided to allow replication by others?

Yes

Reviewer Expertise:

Hand Surgery.

I confirm that I have read this submission and believe that I have an appropriate level of expertise to confirm that it is of an acceptable scientific standard.

# Peer review: Reviewer response for version 1

**DOI:** 10.5256/f1000research.55272.r118393

**Published:** 2022-01-17

**Authors:** Robert Gvozdenovic

**Affiliations:** 1 Department of Orthopedic Surgery, Hand Surgery Unit, Copenhagen University Hospital Gentofte, Hellerup, Denmark

Dear authors, thank you for this submission. Your paper is of high evidence level, well-designed, well-written and easy to read. Nevertheless, I have some comments and questions:

- Despite a good study-design, there might be a potential bias if any differences in fracture-complexity patterns between the groups existed. What if the cast group, for instance, had more complex fracture patterns? Please explain this in details, if only AO 23 A-C1 were included. 
- Does that possibly mean that the results of this study can only have clinical relevance for this particular distal radius fracture pattern? Your conclusion is definitely not in accordance to this assumption. If relevant, correct the conclusion accordingly.
- So, the question is if the brace can also be safely used for more complex distal radius patterns? Add, eventually how many percent of this particular fracture pattern is generally present among all distal radius fracture needing surgery.
- Why this study used only one PROM and not others where minimally important clinical difference (MICD) is existing in the literature (DASH)? If you used them, than the proper sample analysis before the study probably could be foretaken.
- The use of sum median for both the QoR-scores and for opioid use is confusing and make the results not clear. 
- The flowchart needs to be corrected in accordance to the text where you claim that 5 patients did not show up on the follow-up.
- There were not statistically significant difference for the postoperative pain in disfavor of brace. Could this be clinically relevant?
- Mention the eventual cost difference between brace and the cast, if it exists. In some part of the world this question is not insignificant.  

Is the work clearly and accurately presented and does it cite the current literature?

Yes

If applicable, is the statistical analysis and its interpretation appropriate?

Yes

Are all the source data underlying the results available to ensure full reproducibility?

Yes

Is the study design appropriate and is the work technically sound?

Yes

Are the conclusions drawn adequately supported by the results?

Yes

Are sufficient details of methods and analysis provided to allow replication by others?

Yes

Reviewer Expertise:

Hand Surgery.

I confirm that I have read this submission and believe that I have an appropriate level of expertise to confirm that it is of an acceptable scientific standard, however I have significant reservations, as outlined above.

# Response

**Published:** 2022-01-23

**Authors:** Irén Sellbrant

**Affiliations:** Institute of Clinical Science, Sahlgrenska Academy, University of Gothenburg, Sweden

**Reviewer 2 (Robert Gvozdenovic)**

 Dear authors, thank you for this submission. Your paper is of high evidence level, well-designed, well-written and easy to read. Nevertheless, I have some comments and questions:

**Despite a good study-design, there might be a potential bias if any differences in fracture-complexity patterns between the groups existed. What if the cast group, for instance, had more complex fracture patterns? Please explain this in details, if only AO 23 A-C1 were included.**

*Authors Comments:*

*Thank you for this constructive concern. We have made sure from the start that there was no inclusion bias. We have included AO 23, A, B and C1 fractures. These subgroups cover the common distal radius fractures. These fractures are common in this age group, and it is important to be able to make general conclusions. We decided from the very start to exclude C2 and C3 fractures, as they are different type(s) of fracture(s) and call for much more extensive and different treatment. We are therefore confident that there are no major differences in fracture-complexity patterns between the groups. We would also like to mention that this is a prospective randomized study and accordingly, the randomization would take care of minor differences and level out the risk of different cofounders in the different groups. In other words, we have included and randomized fractures of similar complexity levels.*

*This is a prospective randomized study following the inclusion criteria described in the Methods section. Taken together, 6 patients were excluded from analysis, none of these were fracture/surgery related factors while 5 were excluded due to failed block and 1 due to an anatomic deviation.*

**Does that possibly mean that the results of this** **study can only have clinical relevance for this particular distal radius fracture pattern? Your conclusion is definitely not in accordance to this assumption.** **If relevant, correct the conclusion accordingly**.

*Authors Comments:*

*There is only limited information about the use of brace as early immobilisation following open reposition and fixation of distal radius fracture in literature and this is one of the main reasons why this study was performed. As mentioned above, this study included AO 23 A, B and C1 fractures. These are the common distal radius fractures in this age group. There is a high volume, and these fractures are a relatively homogenous group of patients and thus seemingly adequate for inclusion to a randomised study. However, we agree that it is not possible to provide robust comments on the generalisation of the findings. Further studies are needed to confirm its safe use, especially in relation to more complex and complicated fractures (C 2 and C3) as mentioned in the conclusion.*

**So, the question is if the brace can also be safely used for more complex distal radius patterns? Add, eventually how many percent of this particular fracture pattern is generally present among all distal radius fracture needing surgery.**

*Authors Comments:*

*Distal radius fracture (DRF) is one of the most common fractures, with an annual incidence of approximately 25000/year in Sweden. Data from the Swedish fracture register shows that approximately 20.6% of acute DRF undergo surgical fixation primarily and 5.5% secondary surgical fixation. We are aware of that need for open reposition and surgical fixation increases with fracture complexity. We included up to C1 fractures as described above. We do not have explicit national percentage of surgical need for the group studied, but it is without doubt the most common fractures.*

*However, we managed to find statistics for DRF classified to AO 23 C1-3, registered at Sahlgrenska Universityhospital/Mölndals hospital from 2012-2021, see below.*

*C2 and C3, the more complex fractures that not are a part of this study, seems to be 39% of all DRF that needs surgery. That leaves 61% to be the patients in our study.*

DRF class    % of all DRF      % needs surgery

 C1                    12.1                         41.8

 C2                     7.6                          71.1

 C3                     5.5                          84.8

**Why this study used only one PROM and not others where minimally important clinical difference (MICD) is existing in the literature (DASH)? If you used them, then the proper sample analysis before the study probably could be foretaken**.

*Authors Comments:*

*The aim of the study was to assess the quality of recovery with the use of a multi-dimensional validated questionnaire constructed by the Australian group, where Paul Myles is the senior researcher. This quality of recovery questionnaire, which was used in the present study has previously been assessed as an effective scale for studying the recovery following surgery/anesthesia and it includes 5 main domains. And this was the main purpose of the study. It would of course have been of interest to also gain insight to patient reported outcomes, using PROM questionnaire, however, DASH is not optimally suited for evaluation of the early phase following surgery.*

*“Myles et al. defined in a study 2016 updated in 2021, the size of the minimum clinically meaningful difference to the patient. They recommend the value to be 6.0 and the largest difference between the groups that we noted in this study was 4.0. It is, however, important to know that a significant difference in QoR-points doesn´t necessary have to be of clinical value.”* **The use of sum median for both the QoR-scores and for opioid use is confusing and make the results not clear.** *Authors Comments:* *The sum was used to describe that the total score for all 14 questions were analyzed/compared over the time-point studied and between groups. Likewise, the sum describes the **total** amount of opioid use for the 3 postoperative days studied. We have **amended** the text accordingly. Please see the revised manuscript.*

**The flowchart needs to be corrected in accordance to the text where you claim that 5 patients did not show up on the follow-up.**

*Authors Comments:*

*Thank you for important comment; 6 patients **were not included in the analysis** and this is unfortunately described as lost for follow-up in the text. These 6 patients are excluded in the flow chart adequately and the description of exclusion is now corrected in the result section. The wording is, however, incorrect and is now **amended** accordingly.*

**There were not statistically significant difference for the postoperative pain in disfavor of brace. Could this be clinically relevant?**

**Authors Comments:**

*There was a statistically significant difference in QoR pain domain scores at 24 hours postop. The numerical difference of at least 6 has by the score developers, however, shown a clinically relevant difference in domain scores. We found merely a numerical difference of 4. Moreover, we did not see any differences in need for opioid analgesics. This has been further clarified in the method as well as the discussion sections.*

*Myles PS, Myles DB, Galagher W, Chew C, MacDonald N, Dennis A. Minimal Clinically Important Difference for Three Quality of Recovery Scales. Anesthesiology. 2016;125(1):39-45.*

*Myles PS, Myles DB. An Updated Minimal Clinically Important Difference for the QoR-15 Scale. Anesthesiology. 2021;135(5):934-5.* **Mention the eventual cost difference between brace and the cast, if it exists. In some part of the world this question is not insignificant.** *Authors Comments:*​​​​​​​

*We agree that the impact of cost associated to a new intervention must be acknowledged. The direct cost associated to the brace is higher as compared to a cast. However, at our department we routinely apply a brace following the initial 2-week period with cast. The brace applied direct after surgery in this study can thus be used throughout the period. Thus, the net direct cost would not be increased, but possibly decreased. Moreover, the resources for a cast application, and its eventual corrections, will reduce the total cost. It should be acknowledged that all patients were called for a follow-up interview at 4 occasions, enabling patients to raise questions.*

# Peer review: Reviewer response for version 1

**DOI:** 10.5256/f1000research.55272.r86216

**Published:** 2021-06-03

**Authors:** Jakob Walldén

**Affiliations:** 1 Department of Surgical and Perioperative Sciences, Anaesthesiology and Intensive Care Medicine (Sundsvall), Umeå University, Sundsvall, Sweden

It is a well written and structured manuscript evaluating postoperative quality of recovery (QoR) after surgical treatment of distal radius fracture under peripheral blockade. The authors compare immobilization with dorsal splint cast vs. removable brace and as main outcome measures uses self accessed QoR, opioid consumption. The study is an analysis of a subgroup in the RADAR-study (NCT03749174) and the randomization procedure is clearly pre-defined. Underlying data is accessible. The reporting follows CONSORT and statistical methods are appropriate. They find no main differences and concluded that brace is a feasible option. Conclusions are clear and justified.

 However, I have some concerns with the study that needs to be addressed:

 1. The study is a sub-analysis of two out of four arms in the RADAR-study, according to the registration in clinical trials. Please expand the description of the main study in the methods section and justify the decision for this sub-analysis as a separate manuscript.

 Further, how was the quality of recovery in the two other intervention arms? Please comment.

 2 The main outcome measure (QoR-15) is not mentioned in the clinical trial registration. Why? Please explain and also confirm that you had ethical approval to use the QoR-15 as your evaluation tool and that the approval was dated before the inclusion of patients in this prospective study.

 3. Pain assessment with NRS is one of two primary outcome variables in the predefined protocol according to www.clinicaltrials.gov. It is described in the methods section, but results not reported in the manuscript. Please explain/revise. 

 4. Further, why was the occupational therapist evaluation on the 3rd day not included in the analysis? Please explain/discuss.

 5. The use of "sum" in the variables might be confusing for the readers of the paper. The QoR-15 score **is** the sum of all domains and you do not need to add "sum". Please revise all section in the manuscript. For the variable opioid consumption, it is better to use "Total" instead of "sum" (ex: Table 4).

Is the work clearly and accurately presented and does it cite the current literature?

Yes

If applicable, is the statistical analysis and its interpretation appropriate?

Yes

Are all the source data underlying the results available to ensure full reproducibility?

Yes

Is the study design appropriate and is the work technically sound?

Yes

Are the conclusions drawn adequately supported by the results?

Yes

Are sufficient details of methods and analysis provided to allow replication by others?

Yes

Reviewer Expertise:

Anesthesiology, postop-recovery

I confirm that I have read this submission and believe that I have an appropriate level of expertise to confirm that it is of an acceptable scientific standard, however I have significant reservations, as outlined above.

# Response

**Published:** 2021-11-16

**Authors:** Irén Sellbrant

**Affiliations:** Institute of Clinical Science, Sahlgrenska Academy, University of Gothenburg, Sweden

Reviewer Comments:

Reviewer 1 (Jakob Walldén)

 It is a well written and structured manuscript evaluating postoperative quality of recovery (QoR) after surgical treatment of distal radius fracture under peripheral blockade. The authors compare immobilization with dorsal splint cast vs. removable brace and as main outcome measures uses self-accessed QoR, opioid consumption. The study is an analysis of a subgroup in the RADAR-study (NCT03749174) and the randomization procedure is clearly pre-defined. Underlying data is accessible. The reporting follows CONSORT and statistical methods are appropriate. They find no main differences and concluded that brace is a feasible option. Conclusions are clear and justified.

 However, I have some concerns with the study that needs to be addressed:

 1. The study is a sub-analysis of two out of four arms in the RADAR-study, according to the registration in clinical trials. Please expand the description of the main study in the methods section and justify the decision for this sub-analysis as a separate manuscript.

***Authors Comments:***

***The main RADAR-study was performed to assess different anaesthetic techniques and their impact on postoperative pain (NRS) and opioid consumption during the first 3 postoperative days.***

***The RADAR-study arms assessing the impact of immobilization techniques was aimed to compare traditional cast with a flexible brace already as early fixation, directly post-surgery. Our interest was to assess whether the brace could improve the day case perioperative course, speeding up the perioperative time events and reducing early postoperative discomfort commonly associated to the cast fixation. The occupational therapist, also PhD-student, had the goal to assess the effects on the rehabilitation, need for unplanned visits due to cast issues and the functional outcome for up to 12-month post-surgery.***

 Further, how was the quality of recovery in the two other intervention arms? Please comment.

***Authors Comments:***

***The main object for the “main RADAR-study” was pain and opioid requirement, assessing the risk for rebound pain following block resolution and subsequent increased need for  opioid analgesic during the intermediate postoperative course. The study aims were impact of the pain 24 hours post-surgery and during the first 3 postoperative days and opioid requirement during the same period, PONV/PDNV, time events, time to discharge and unplanned health contacts. We did unfortunately not focus on the quality of recovery in that part of the study.***

 2. The main outcome measure (QoR-15) is not mentioned in the clinical trial registration. Why?

 Please explain and also confirm that you had ethical approval to use the QoR-15 as your evaluation tool and that the approval was dated before the inclusion of patients in this prospective study.

***Authors Comments:***

***The ethical review protocol was approved May 31st 2018 (as described in the method section) and contains QoR-15,*** ***The Clinical trial Registration was unfortunately not fully aligned with the ethical application, but is now updated accordingly*** **clinicaltrials.gov (NCT03749174).**

 3. Pain assessment with NRS is one of two primary outcome variables in the predefined protocol according to www.clinicaltrials.gov. It is described in the methods section, but results not reported in the manuscript. Please explain/revise.

***Authors Comments:***

***The primary of this part of the study was the QoR-15 questionnaire, time events during the perioperative course and the opioid consumption. We assessed pain in this subgroup by the QoR questions that showed a pain difference at 24 hours in favor for the traditional cast.***

 4. Further, why was the occupational therapist evaluation on the 3rd day not included in the analysis? Please explain/discuss.

***Authors Comments:***

***The occupational therapist follow-up is still not completed (partly because of the COVID-19 Pandemic) and will commence up to 12 months’ post-surgery.*** ***The more in depth assessment of wrist function will hopefully, although delayed, be performed by the occupational therapists during coming autumn and winter.*******

 5. The use of "sum" in the variables might be confusing for the readers of the paper. The QoR-15 score is the sum of all domains and you do not need to add "sum". Please revise all section in the manuscript. For the variable opioid consumption, it is better to use "Total" instead of "sum" (ex: Table 4).

***Authors Comments:***

***Corrected accordingly.***

# Response

**Published:** 2022-01-23

**Authors:** Irén Sellbrant

**Affiliations:** Institute of Clinical Science, Sahlgrenska Academy, University of Gothenburg, Sweden

**Reviewer 1 (Jakob Walldén)**

 It is a well written and structured manuscript evaluating postoperative quality of recovery (QoR) after surgical treatment of distal radius fracture under peripheral blockade. The authors compare immobilization with dorsal splint cast vs. removable brace and as main outcome measures uses self-accessed QoR, opioid consumption. The study is an analysis of a subgroup in the RADAR-study (NCT03749174) and the randomization procedure is clearly pre-defined. Underlying data is accessible. The reporting follows CONSORT and statistical methods are appropriate. They find no main differences and concluded that brace is a feasible option. Conclusions are clear and justified. However, I have some concerns with the study that needs to be addressed:

**1.** **The study is a sub-analysis of two out of four arms in the RADAR-study, according to the** **registration in clinical trials. Please expand the description of the main study in the methods** **section and justify the decision for this sub-analysis as a separate manuscript**.

*Authors Comments:*

*The main RADAR-study was performed to assess different anaesthetic techniques and their impact on postoperative pain (NRS) and opioid consumption during the first 3 postoperative days. The main RADAD-study is now further clarified in the method section and aligned to the Clinical Trial registration.*

**

*The RADAR-study arms assessing the impact of immobilization techniques was aimed to compare traditional cast with a flexible brace already as early fixation, directly post-surgery. This is a separate intervention. Our hypothesis was that brace fixation direct after surgery could improve the day case perioperative course, speeding up the perioperative time events and reducing early postoperative discomfort commonly associated to the cast fixation and facilitate activities of daily living. The occupational therapist, also PhD-student, had the goal to assess the effects on the rehabilitation, need for unplanned visits due to cast issues and the functional outcome for up to 12-month post-surgery.*

**Further, how was the quality of recovery in the two other intervention arms? Please comment.**

*Authors Comments:*

*The object for the “main RADAR-study” was pain and opioid requirement, assessing the risk for rebound pain following block resolution and subsequent increased need for opioid analgesic during the intermediate postoperative course. The study aims were impact of the pain 24-hours post-surgery and during the first 3 postoperative days and opioid requirement during the same period, PONV/PDNV, time events, time to discharge and unplanned health contacts. We did unfortunately not focus on the quality of recovery in that part of the study.*

**2.** **The main outcome measure (QoR-15) is not mentioned in the clinical trial registration. Why?** **Please explain and also confirm that you had ethical approval to use the QoR-15 as your** **evaluation tool and that the approval was dated before the inclusion of patients in this prospective** **study.**

*Authors Comments:*

*The ethical review protocol was approved May 31:st 2018 (as described in the method section) and contains QoR-15.* *The Clinical trials Registration was not fully aligned with the ethical application, but is now updated accordingly* *clinicaltrials.gov (NCT03749174).*

**3.** **Pain assessment with NRS is one of two primary outcome variables in the predefined protocol** **according to www.clinicaltrials.gov. It is described in the methods section, but results not reported in the manuscript. Please explain/revise.**

*Authors Comments:*

*The primary of this part of the study was the QoR-15 questionnaire, time events during the perioperative course and the opioid consumption. We assessed pain in this subgroup by the QoR questions that showed a pain difference at 24 hours in favor for the traditional cast. However, the numerical difference was less than what has been shown as a clinical relevant difference by Myles et al. (references). This is now clarified in the method as well as in the discussion section.*

******

**4.** **Further, why was the occupational therapist evaluation on the 3rd day not included in the** **analysis? Please explain/discuss.**

*Authors Comments:*

*The occupational therapist follow-up is still not completed (partly because of the Covid-Pandemic) and will commence up to 12 months’ post-surgery.* *The more in-depth assessment of wrist function will hopefully, although, delayed be performed by the occupational therapists during coming autumn and winter.*

**5.** **The use of "sum" in the variables might be confusing for the readers of the paper. The QoR-15** **score is the sum of all domains and you do not need to add "sum". Please revise all section in the** **manuscript. For the variable opioid consumption, it is better to use "Total" instead of "sum" (ex: Table 4).**

*Authors Comments:*

*Corrected accordingly.*
